# Risk factor analysis and development of a risk prediction model for facial pressure injury associated with noninvasive positive pressure ventilation in patients with chronic obstructive pulmonary disease based on symptom science theory

**DOI:** 10.3389/fmed.2026.1897955

**Published:** 2026-10-02

**Authors:** Chen Cheng, Lulu Cui, Changhai Wang, Yujie Zhang, Jun Ge, Jinmei Peng, Bin Zhang, Rong Zhao, Duoduo Qiao, Xiaoyun Chen, Yaojun Zhang, Pei Zhang, Manhong Zhang, Yan Wang, Xuling Gao, Changchun Song, Qiankun Liu

**Affiliations:** 1 Department of Nursing, Bengbu First People’s Hospital, Bengbu, Anhui, China; 2 Department of Nursing, Bengbu Third People’s Hospital, Bengbu, Anhui, China

**Keywords:** chronic obstructive pulmonary disease, facial pressure injury, machine learning, noninvasive positive pressure ventilation, prediction model

## Abstract

**Background:**

Noninvasive positive pressure ventilation is a key treatment for acute exacerbations of chronic obstructive pulmonary disease and is associated with a facial pressure injury incidence of 20% to 30%. However, current risk assessment still relies on generic tools such as the Braden scale, and there is a lack of risk prediction instruments that incorporate ventilation related respiratory mechanical parameters.

**Objective:**

Based on the Symptom Science Model 2.0, this study systematically examined factors associated with noninvasive positive pressure ventilation related facial pressure injury in patients with chronic obstructive pulmonary disease and developed and temporally validated a risk prediction model to provide a quantitative tool for early clinical warning and risk stratified prevention.

**Methods:**

From January 2020 to February 2025, we collected clinical data from 390 patients with chronic obstructive pulmonary disease receiving noninvasive positive pressure ventilation. Participants were divided by enrollment time into a training cohort of 273 patients enrolled between January 2020 and September 2023 for model development and a temporal validation cohort of 117 patients enrolled between October 2023 and February 2025 for model validation. Facial pressure injury was the outcome. Candidate predictors were screened using univariate analysis and least absolute shrinkage and selection operator regression, followed by multivariable logistic regression for model development, and 17 machine learning models were compared using five fold cross validation. Model performance was assessed using the area under the receiver operating characteristic curve, calibration curves, and decision curve analysis, and DeLong's test was used to compare area under the curve values. The selected model was further interpreted and visualized using SHAP.

**Results:**

The overall incidence of noninvasive positive pressure ventilation–related facial pressure injury was 23.33%. Multivariable logistic regression identified leak volume >40 L/min, higher inspiratory pressure, total ventilation time >100 h, single session duration ≥4 h, serum albumin <35 g/L, and a history of noninvasive ventilation use as independent risk factors, whereas BMI ≥18.5 kg/m^2^ was a protective factor. Among the 17 models, the traditional and parsimonious logistic regression model was recommended, achieving an AUC of 0.785 (95% CI: 0.702–0.868) in the temporal validation cohort; calibration and decision curve analyses also indicated good agreement and favorable clinical net benefit. SHAP analysis showed that serum albumin, total ventilation time, and inspiratory pressure contributed most to the model.

**Conclusion:**

Based on the Symptom Science Model 2.0, we developed and temporally validated a risk prediction model that integrates noninvasive positive pressure ventilation–specific mechanical parameters. This model may serve as a quantitative tool for early identification of high risk patients and potentially help reduce the incidence of noninvasive positive pressure ventilation–related facial pressure injury.

## 1 Introduction

Chronic obstructive pulmonary disease (COPD) is a common chronic respiratory disorder characterized by persistent airflow limitation (1)and has become a major global public health challenge (2). According to the Global Burden of Disease Study, COPD is currently the third leading cause of death worldwide (3). In China, a large-scale epidemiological study reported that the prevalence of COPD among adults aged 40 years and older is approximately 8.6%, and it continues to increase with population aging (4). For patients with acute exacerbation of COPD (AECOPD) complicated by respiratory failure, noninvasive positive pressure ventilation (NPPV) is an important treatment to improve gas exchange and reduce the need for endotracheal intubation and mortality (5), and it is recommended as a first-line therapy by both domestic and international guidelines (6). However, prolonged use of NPPV may lead to device-related complications due to mask compression and local moisture-related irritation (7), among which facial pressure injury is the most common and clinically consequential.

Preventing NPPV-related facial pressure injury can be challenging because mask pressure may be difficult to relieve during ongoing ventilation. Tightening straps to reduce air leaks may increase pressure over the nasal bridge and compromise local tissue perfusion (8). The incidence of NPPV-related facial pressure injury may reach 25%, most commonly involving the nasal bridge, forehead, and ears (9, 10). Once skin breakdown occurs, pain, secondary infection, reduced treatment tolerance, and even discontinuation of NPPV may follow (11). In patients with COPD, interruption of NPPV may increase the risk of ventilation failure, endotracheal intubation, prolonged hospitalization, and higher medical costs (11). Therefore, accurate risk stratification is essential for early prevention and targeted nursing intervention.

Current risk assessment for NPPV-related facial pressure injury still largely relies on generic tools such as the Braden scale (12)or traditional risk factors, including age, diabetes, and ventilation duration (13). However, these approaches may not fully capture device-specific biomechanical exposures during NPPV. The fourth edition of the international pressure ulcer guideline emphasizes the need to incorporate device-related risk factors into risk assessment (14). Etiological models also suggest that pressure injury occurs when external mechanical loads exceed tissue tolerance (15). During NPPV, parameters such as IPAP and mask leak volume can influence pressure distribution at the skin–mask interface and contribute to injury development (16). Therefore, an NPPV-specific prediction model incorporating ventilation-related parameters may support earlier and more individualized risk assessment.

Machine learning (ML) provides a powerful approach for integrating complex and nonlinear clinical data and has demonstrated performance beyond traditional methods in prediction tasks in critical care (17, 18). However, ML models that integrate respiratory mechanical parameters to predict NPPV-related facial pressure injury in patients with COPD are still lacking. In current clinical prediction research, feature selection and variable inclusion in ML models are often primarily data driven and insufficiently guided by an explicit theoretical framework, which may lead to indiscriminate variable accumulation and limited interpretability, thereby reducing usability and generalizability in nursing decision making.

To address these limitations, we propose to use the SSM 2.0 as the theoretical framework. SSM 2.0 emphasizes starting from “complex symptoms” and integrating phenotypic characteristics, biobehavioral factors, and clinical application (19), while also considering patient experience and the social determinants of health to more comprehensively explain symptom occurrence and outcome differences. Accordingly, we conceptualize NPPV-related facial pressure injury as an important device-related complication during therapy. We will systematically identify candidate predictors across multiple domains, including baseline characteristics and comorbidities, nutritional and physiological indicators, key mechanical parameters of noninvasive ventilation, and patient comfort and cooperation. We will develop and compare multiple ML models and apply interpretability analyses to identify key driving factors. The findings will support early clinical warning and risk-stratified prevention, reduce the incidence of facial pressure injury, and improve the safety and quality of respiratory support. In addition, this study may enhance clinicians’ understanding and standardized use of ventilation mechanical parameters and inform future smart nursing practice.

## 2 Methods

### 2.1 Study participants

This retrospective study collected clinical data from 390 patients with chronic obstructive pulmonary disease who received noninvasive positive pressure ventilation between January 2020 and February 2025 at Bengbu First People's Hospital and Bengbu Third People's Hospital. Based on the enrollment period, 273 patients enrolled from January 2020 to September 2023 were assigned to the training cohort, and 117 patients enrolled from October 2023 to February 2025 were assigned to the validation cohort. The training cohort was used for risk factor analysis and prediction model development, while the validation cohort was used to evaluate the model's discriminative ability and calibration.

Inclusion criteria were as follows: (1) meeting the diagnostic criteria for COPD according to the 2024 Global Initiative for Chronic Obstructive Lung Disease (GOLD) report (20); (2) requiring NPPV during hospitalization, with complete and traceable ventilator monitoring parameters (e.g., IPAP, leak volume, and duration of use); and (3) complete general admission information, laboratory test results, and nursing records. Exclusion criteria were as follows: (1) presence of facial pressure injury before initiation of NPPV; (2) obvious facial deformity, recent facial trauma, or facial surgery affecting mask fitting; (3) confirmed allergy to mask materials or fixation dressings that precluded continuation of NPPV; and (4) substantial missing key variables or inability to determine the outcome.

### 2.2 Ascertainment of facial pressure injury

Facial pressure injury was identified according to the second update of the 2019 International Clinical Practice Guideline (21)and confirmed by the responsible nurses based on nursing records and skin assessment findings.

### 2.3 Selection of predictors

Using the Symptom Science Model 2.0 as the theoretical framework, we selected and organized candidate predictors in a stratified manner. In this study, NPPV-related facial pressure injury was regarded as a complex symptom outcome. Candidate predictors were mapped onto four SSM 2.0–informed domains: phenotypic characteristics, biobehavioral factors, patient-centered experience, and social determinants of health. Phenotypic characteristics represented baseline biological susceptibility; biobehavioral factors reflected treatment exposure, physiological responses, and medication-related influences; patient-centered experience captured subjective psychological status; and social determinants of health represented educational background that may affect health literacy and preventive behaviors. The detailed mapping of variables to SSM 2.0 domains is shown in Table 1.

**Table 1:** Classification of candidate predictors for NPPV-related facial pressure injury based on the symptom science model 2.0.

SSM 2.0 domain	Candidate predictors	Coding
Outcome	NPPV-related facial pressure injury	0 = no, 1 = yes
Phenotypic characteristics	Age	Continuous or categorized according to study design
	Sex	0 = female, 1 = male
	BMI	0 = <18.5 kg/m^2^, 1 = 18.5–24.9 kg/m^2^, 2 = ≥25 kg/m^2^
	Diabetes	0 = no, 1 = yes
	Hypertension	0 = no, 1 = yes
	History of noninvasive ventilation use	0 = no, 1 = yes
	Serum albumin	0 = ≥35 g/L, 1 = <35 g/L
	Hemoglobin	Continuous variable
	Triglycerides	Continuous variable
Biobehavioral factors	Inspiratory positive airway pressure	Continuous variable
	Leak volume	0 = 0–19 L/min, 1 = 20–39 L/min, 2 = ≥40 L/min
	Pressure rise time	Continuous variable
	Total duration of NPPV use	0 = <50 h, 1 = 50–100 h, 2 = >100 h
	Single-session duration	0 = <4 h, 1 = ≥4 h
	Oxygenation index	0 = ＞300 mmHg, 1 = 200–300 mmHg, 2 = 100–200 mmHg, 3 = <100 mmHg
	Body temperature	0 = <37.5 °C, 1 = ≥37.5 °C
	Corticosteroid use	0 = no, 1 = yes
	Vasoactive drug use	0 = no, 1 = yes
	Antibiotic use	0 = no, 1 = yes
Patient-centered experience	Anxiety status	0 = no, 1 = yes
Social determinants of health	Educational attainment	0 = illiterate, 1 = 1–6 years, 2 = 7–12 years, 3 = >12 years

### 2.4 Statistical analysis

Statistical analyses and model development were performed using SPSS 26.0, R version 4.5.2, and Python version 3.12.7. Continuous variables were first assessed for normality. Normally distributed variables are presented as mean ± standard deviation and were compared using the independent-samples t test, whereas non-normally distributed variables are presented as median and interquartile range and were compared using the Mann–Whitney U test. Participants enrolled between January 2020 and September 2023 were assigned to the training cohort, and those enrolled between October 2023 and February 2025 were assigned to a temporally separated validation cohort. With facial pressure injury as the outcome, variables with *P* < 0.05 in univariate analyses were entered into least absolute shrinkage and selection operator regression for feature selection. Multicollinearity among candidate predictors was assessed using the variance inflation factor, with VIF < 5 indicating no substantial collinearity. Selected variables were then included in a multivariable logistic regression model. In the training cohort, 17 machine learning models were developed and compared using five-fold cross-validation with a fixed random seed to improve reproducibility. When required by the algorithm, continuous variables were standardized and categorical variables were transformed using one-hot encoding. The temporally separated validation cohort was not used for feature selection, model training, or parameter selection, and was reserved only for final model evaluation. Based on model performance, stability, interpretability, and clinical applicability, logistic regression was selected as the final model. For the final logistic regression model, continuous predictors were entered on their original scales. Categorical predictors were transformed using OneHotEncoder, with the first category dropped as the reference category. The classifier was fitted with the following parameters: solver = “liblinear”, penalty = “l1”, C = 1.0, and max_ite*r* = 1000, with a fixed random_state for reproducibility. Precision–recall curves, calibration curves, and decision curve analyses were generated for the training and temporal validation cohorts to evaluate discrimination, calibration, and clinical net benefit. The DeLong test was used to compare AUCs across models in the training and validation cohorts. The final logistic regression model was further interpreted using SHAP, with feature importance bar plots and beeswarm plots produced. All tests were two-sided, and *P* < 0.05 was considered statistically significant.

## 3 Results

### 3.1 Baseline characteristics of the training and validation cohorts

A total of 390 patients with chronic obstructive pulmonary disease receiving noninvasive positive pressure ventilation were included, comprising 273 in the training cohort and 117 in the validation cohort (Table 2). Both cohorts were predominantly male. Leak volume was mainly in the 0–39 L/min range, BMI was most commonly 18.5–24.9 kg/m^2^, and the oxygenation index was largely between 200 and 300 mmHg. Regarding ventilation-related variables, total duration of use was predominantly <50 h and single-session duration was mostly <4 h; few patients had a body temperature ≥37.5 °C, and approximately one-third had serum albumin levels <35 g/L (34.07% in the training cohort and 31.62% in the temporal validation cohort). The distributions of diabetes and hypertension were similar between cohorts; most patients did not receive vasoactive drugs, whereas antibiotic use was relatively common. Educational attainment was mainly ≤12 years; most patients had no history of noninvasive ventilation, and anxiety was present in about one-quarter. The median age was 74 years in both cohorts, and the median IPAP was 12 cm H₂O. The incidence of facial pressure injury was 23.81% in the training cohort and 22.22% in the validation cohort.

**Table 2:** Characteristics of COPD patients receiving noninvasive positive pressure ventilation in the training and validation cohorts.

Variable	Category	Validation cohort (*n* = 117)	Training cohort (*n* = 273)
Sex	Female	45 (38.46%)	107 (39.19%)
	Male	72 (61.54%)	166 (60.81%)
Leak volume	0-19L/min	59 (50.43%)	131 (47.99%)
	20-39L/min	52 (44.44%)	123 (45.05%)
	＞40L/min	6 (5.13%)	19 (6.96%)
BMI	＜18.5 kg/m^2^	23 (19.66%)	55 (20.15%)
	18.5-24.9 kg/m^2^	64 (54.70%)	144 (52.75%)
	≥25 kg/m^2^	30 (25.64%)	74 (27.11%)
Oxygenation index	＞300mmHg	30 (25.64%)	75 (27.47%)
	200-300mmHg	47 (40.17%)	106 (38.83%)
	100-200mmHg	30 (25.64%)	69 (25.27%)
	＜100mmHg	10 (8.55%)	23 (8.42%)
Total usage duration	＜50h	68 (58.12%)	153 (56.04%)
	50-100h	16 (13.68%)	48 (17.58%)
	＞100h	33 (28.21%)	72 (26.37%)
Duration per session	＜4h	73 (62.39%)	172 (63.00%)
	≥4h	44 (37.61%)	101 (37.00%)
Temperature	＜37.5℃	106 (90.60%)	235 (86.08%)
	≥37.5℃	11 (9.40%)	38 (13.92%)
Serum albumin	≥35 g/L	80 (68.38%)	180 (65.93%)
	＜35 g/L	37 (31.62%)	93 (34.07%)
Diabetes	No	91 (77.78%)	205 (75.09%)
	Yes	26 (22.22%)	68 (24.91%)
Hypertension	No	46 (39.32%)	114 (41.76%)
	Yes	71 (60.68%)	159 (58.24%)
Corticosteroid use	No	19 (16.24%)	39 (14.29%)
	Yes	98 (83.76%)	234 (85.71%)
Vasoactive drugs	Not used	99 (84.62%)	231 (84.62%)
	Used	18 (15.38%)	42 (15.38%)
Antibiotic use	No	20 (17.09%)	46 (16.85%)
	Yes	97 (82.91%)	227 (83.15%)
Education level	Illiterate	26 (22.22%)	60 (21.98%)
	≤6years	43 (36.75%)	109 (39.93%)
	7-12years	42 (35.90%)	92 (33.70%)
	＞12years	6 (5.13%)	12 (4.40%)
History of NIV use	No	86 (73.50%)	171 (62.64%)
	Yes	31 (26.50%)	102 (37.36%)
Anxiety	No	90 (76.92%)	204 (74.73%)
	Yes	27 (23.08%)	69 (25.27%)
Age（years）		74(Q1:66.0,Q3:81.0)	74(Q1:67.0,Q3:80.0)
IPAP,（cm H₂O）		12(Q1:11.0,Q3:14.0)	12(Q1:12.0,Q3:16.0)
Facial pressure injury	Yes	26 (22.22%)	65 (23.81%)
	No	91 (77.78%)	208 (76.19%)

### 3.2 Univariate analysis

Table 3 shows that, in the training cohort, significant differences were observed between the no pressure injury group and the pressure injury group in leak volume, BMI, total usage duration, duration per session, serum albumin, history of noninvasive ventilation use, and IPAP (all *P* < 0.05). In contrast, no significant between-group differences were found for sex, oxygenation index, body temperature, diabetes, hypertension, corticosteroid use, vasoactive drug use, antibiotic use, educational attainment, anxiety, age, pressure rise time, triglycerides, or hemoglobin. In terms of distributional patterns, the pressure injury group tended to have higher leak volume and a higher proportion of low BMI. Regarding ventilatory exposure, total usage duration >100 h and duration per session ≥4 h were more common in the pressure injury group.

**Table 3:** Univariate analysis of factors associated with facial pressure injury in the training cohort.

Variable	Category	No pressure injury (*n* = 208)	Pressure injury (*n* = 65)	Statistic	P
**Sex**	Female	84 (40.38%)	23 (35.38%)	*χ*^2^ = 0.331	0.565
	Male	124 (59.62%)	42 (64.62%)		
**Leak volume**	0-19L/min	115 (55.29%)	16 (24.62%)	χ^2^ = 42.548	＜0.001
	20-39L/min	89 (42.79%)	34 (52.31%)		
	＞40L/min	4 (1.92%)	15 (23.08%)		
**BMI**	＜18.5 kg/m^2^	30 (14.42%)	25 (38.46%)	χ^2^ = 18.016	＜0.001
	18.5-24.9 kg/m^2^	119 (57.21%)	25 (38.46%)		
	≥25 kg/m^2^	59 (28.37%)	15 (23.08%)		
**Oxygenation index**	＞300mmHg	59 (28.37%)	16 (24.62%)	χ^2^ = 2.772	0.428
	200-300mmHg	80 (38.46%)	26 (40.00%)		
	100-200mmHg	49 (23.56%)	20 (30.77%)		
	＜100mmHg	20 (9.62%)	3 (4.62%)		
**Total usage duration**	＜50h	141 (67.79%)	12 (18.46%)	χ^2^ = 72.924	＜0.001
	50-100h	38 (18.27%)	10 (15.38%)		
	＞100h	29 (13.94%)	43 (66.15%)		
**Duration per session**	＜4h	155 (74.52%)	17 (26.15%)	χ^2^ = 47.647	＜0.001
	≥4h	53 (25.48%)	48 (73.85%)		
**Temperature**	＜37.5℃	180 (86.54%)	55 (84.62%)	χ^2^ = 0.034	0.853
	≥37.5℃	28 (13.46%)	10 (15.38%)		
**Serum albumin**	≥35 g/L	163 (78.37%)	17 (26.15%)	χ^2^ = 57.804	＜0.001
	＜35 g/L	45 (21.63%)	48 (73.85%)		
**Diabetes**	No	155 (74.52%)	50 (76.92%)	χ^2^ = 0.051	0.821
	Yes	53 (25.48%)	15 (23.08%)		
**Hypertension**	No	83 (39.90%)	31 (47.69%)	χ^2^ = 0.936	0.333
	Yes	125 (60.10%)	34 (52.31%)		
**Corticosteroid use**	No	34 (16.35%)	5 (7.69%)	χ^2^ = 2.363	0.124
	Yes	174 (83.65%)	60 (92.31%)		
**Vasoactive drugs**	Not used	177 (85.10%)	54 (83.08%)	χ^2^ = 0.039	0.844
	Used	31 (14.90%)	11 (16.92%)		
**Antibiotic use**	No	38 (18.27%)	8 (12.31%)	χ^2^ = 0.867	0.352
	Yes	170 (81.73%)	57 (87.69%)		
**Education level**	Illiterate	44 (21.15%)	16 (24.62%)	χ^2^ = 2.931	0.402
	≤6years	84 (40.38%)	25 (38.46%)		
	7-12years	73 (35.10%)	19 (29.23%)		
	＞12years	7 (3.37%)	5 (7.69%)		
**History of NIV use**	No	152 (73.08%)	19 (29.23%)	χ^2^ = 38.830	＜0.001
	Yes	56 (26.92%)	46 (70.77%)		
**Anxiety**	No	153 (73.56%)	51 (78.46%)	χ^2^ = 0.398	0.528
	Yes	55 (26.44%)	14 (21.54%)		
**Age (years)**		74.0(Q1:67.75,Q3:80.0)	72.0(Q1:64.0,Q3:79.0)	z = 0.753	0.451
**IPAP (cm H₂O)**		12.0(Q1:11.0,Q3:14.0)	16.0(Q1:13.0,Q3:18.0)	z = -6.302	＜0.001
**Pressure rise time (s)**		0.15(Q1:0.15,Q3:0.2)	0.15(Q1:0.15,Q3:0.2)	z = -0.360	0.672
**Triglycerides (mmol/L)**		0.89(Q1:0.708,Q3:1.21)	0.95(Q1:0.76,Q3:1.37)	z = -1.416	0.157
**Hemoglobin (g/L)**		131.0(Q1:116.0,Q3:147.0)	129.0(Q1:115.0,Q3:149.0)	z = -0.166	0.869

### 3.2 Feature selection

Variables that were statistically significant in the univariate analysis (*P* < 0.05) were entered into least absolute shrinkage and selection operator (LASSO) regression for further feature selection. After determining the optimal penalty parameter by cross-validation, seven predictors with nonzero coefficients were retained: leak volume, total usage duration, BMI, duration per session, serum albumin, history of NIV use, and IPAP (Figure 1). Multicollinearity was then assessed for these seven predictors, and all variance inflation factors were <5. Therefore, no substantial multicollinearity was detected, and the selected predictors were included in the subsequent multivariable analysis.

**Figure 1:**
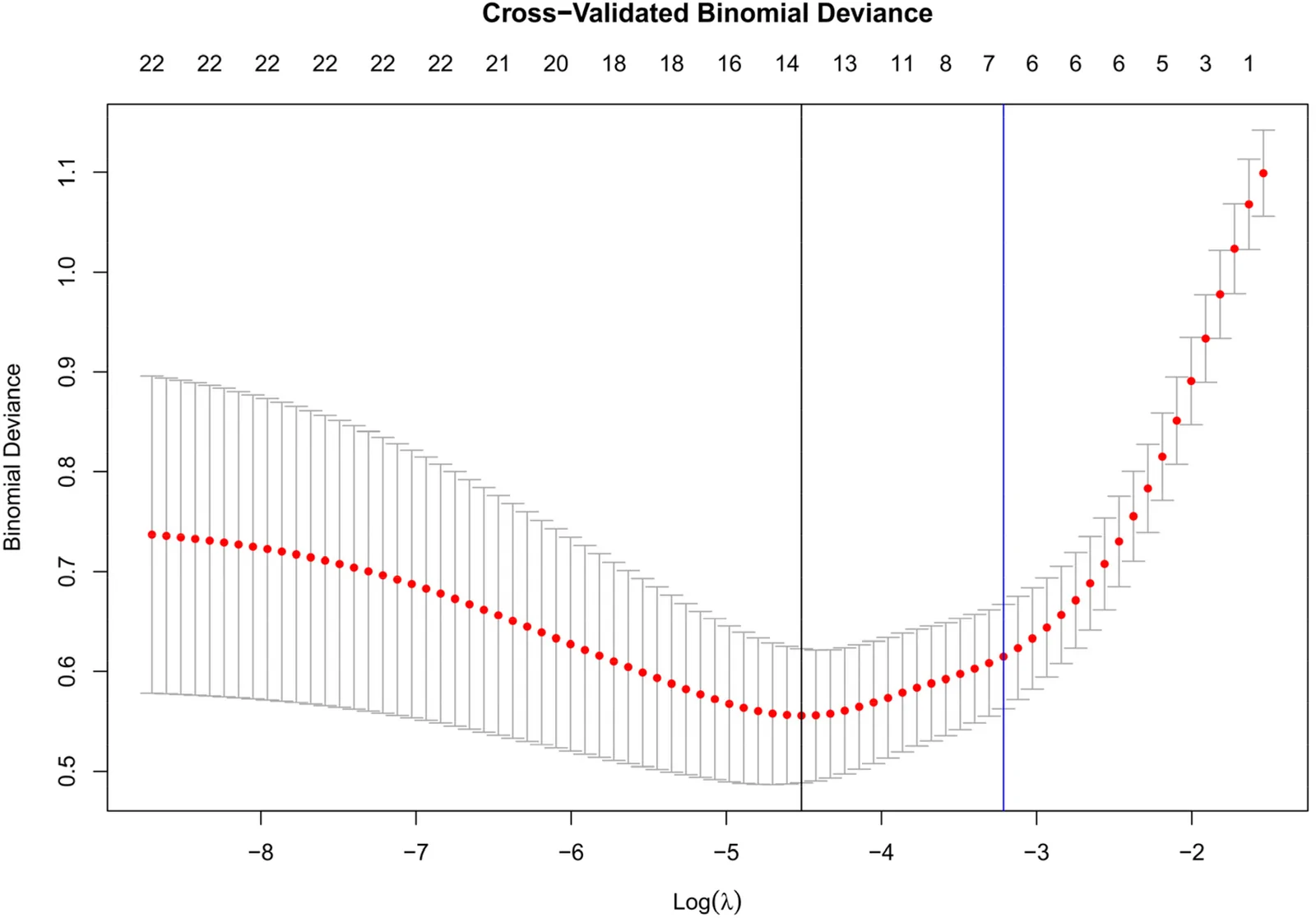
LASSO feature selection for facial pressure injury in patients with chronic obstructive pulmonary disease receiving noninvasive positive pressure ventilation.

### 3.3 Multivariable analysis

The seven predictors selected by LASSO were entered into a multivariable logistic regression model. As shown in Table 4, leak volume >40 L/min (OR 7.134, 95% CI 1.017–50.064), higher IPAP (OR 2.852, 95% CI 1.649–4.935), total usage duration >100 h (OR 9.406, 95% CI 2.922–30.279), duration per session ≥4 h (OR 8.305, 95% CI 2.879–23.955), serum albumin <35 g/L (OR 7.788, 95% CI 2.747–22.080), and a history of NIV use (OR 2.997, 95% CI 1.034–8.683) were all associated with an increased risk of facial pressure injury. Compared with BMI <18.5 kg/m^2^, BMI 18.5–24.9 kg/m^2^ (OR 0.143, 95% CI 0.040–0.516) and BMI ≥25 kg/m^2^ (OR 0.195, 95% CI 0.045–0.848) were associated with a lower risk of facial pressure injury. In addition, compared with total usage duration <50 h, total usage duration of 50–100 h (OR 0.992, 95% CI 0.225–4.378) was not statistically significant.

**Table 4:** Multivariable analysis of facial pressure injury in patients with chronic obstructive pulmonary disease receiving noninvasive positive pressure ventilation.

Predictors	*β*	SE	Odds Ratio	95%CI	P
Leak volume(Reference 0-19L/min)					
20-39L/min	0.538	0.535	1.712	0.600-4.884	0.314
＞40L/min	1.965	0.994	7.134	1.017-50.064	0.048
IPAP(per 1 cm H₂O increase)	1.048	0.280	2.852	1.649-4.935	<0.001
BMI(Reference group:<18.5 kg/m^2^)					
18.5-24.9 kg/m^2^	-1.946	0.655	0.143	0.040-0.516	0.003
≥25 kg/m^2^	-1.634	0.749	0.195	0.045-0.848	0.029
Total usage duration(Reference group:<50 h)					
50-100h	-0.008	0.757	0.992	0.225-4.378	0.992
＞100h	2.241	0.596	9.406	2.922-30.279	<0.001
Duration per session(Reference group:<4 h)					
≥4h	2.117	0.541	8.305	2.879-23.955	<0.001
Serum albumin(Reference group:≥35 g/L)					
<35 g/L	2.053	0.532	7.788	2.747-22.080	<0.001
History of NIV use(Reference group:No)					
Yes	1.097	0.543	2.997	1.034-8.683	0.043

### 3.4 Model development and validation

Seventeen machine learning prediction models were developed in this study, and ROC curves, calibration curves, and decision curve analyses were generated in both the training and validation cohorts to comprehensively evaluate model performance. As shown in Figure 2, the ROC curves of the models were generally similar across the two cohorts. AUCs were overall higher in the training cohort than in the validation cohort, with validation AUCs mainly around 0.70, indicating good discrimination and relatively small differences among models. Figure 3 presents the multi-model calibration curves; most curves were close to the ideal calibration line, suggesting acceptable agreement between predicted and observed probabilities, although some deviations were observed in certain probability ranges. The precision–recall curves in Figure 4 indicate that all models showed reasonable discriminative ability, with most curves trending toward the upper-left region, reflecting good classification performance. In addition, the differences in AUC between the training and validation cohorts were small, suggesting good generalizability. The decision curve analysis in Figure 5 shows that most models achieved a positive net benefit across a wide range of threshold probabilities, supporting their potential clinical utility.

**Figure 2:**
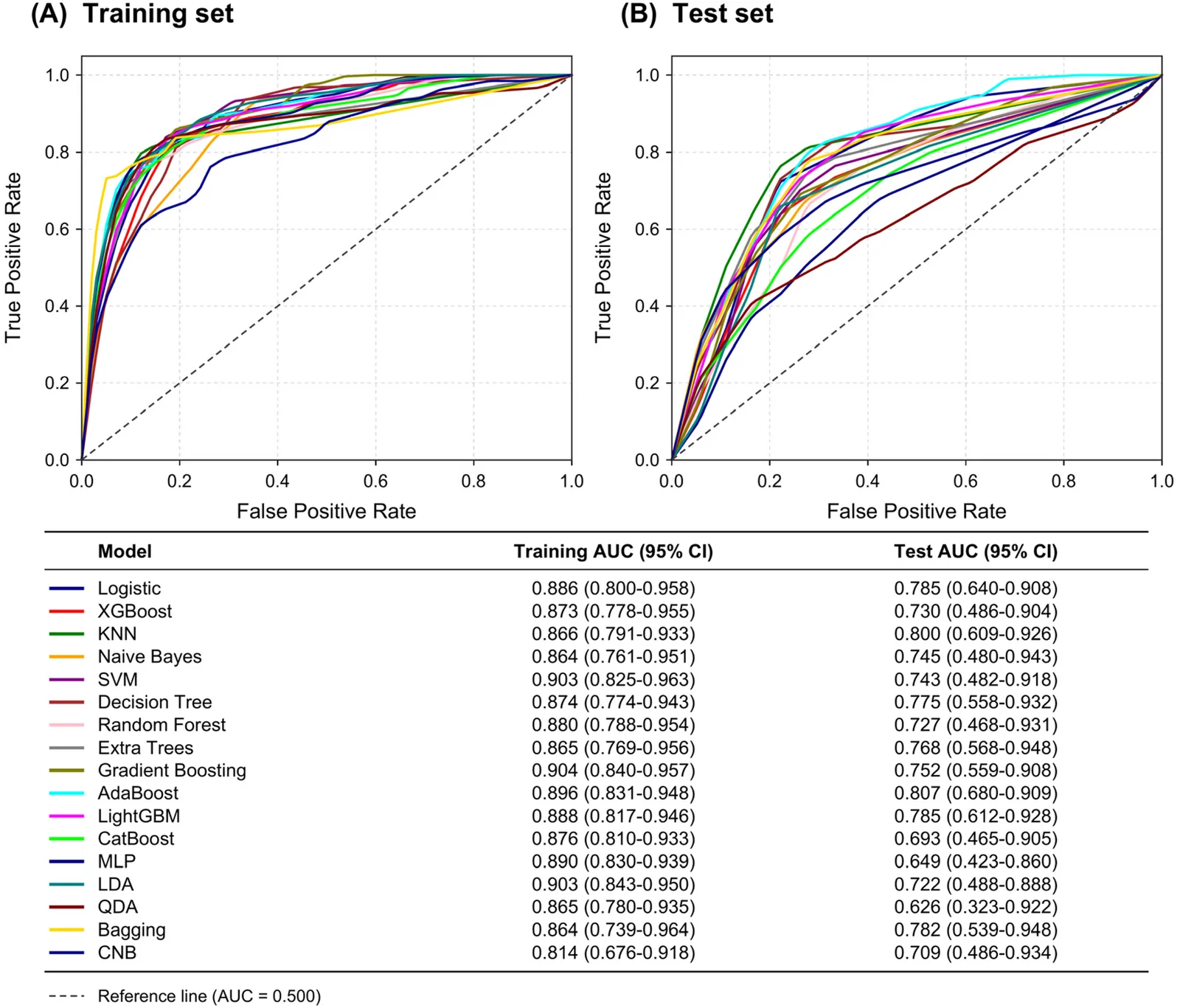
ROC curves of machine learning–based prediction models for facial pressure injury in patients with chronic obstructive pulmonary disease receiving noninvasive positive pressure ventilation. **(A)** Training set and **(B)** Test set.

**Figure 3:**
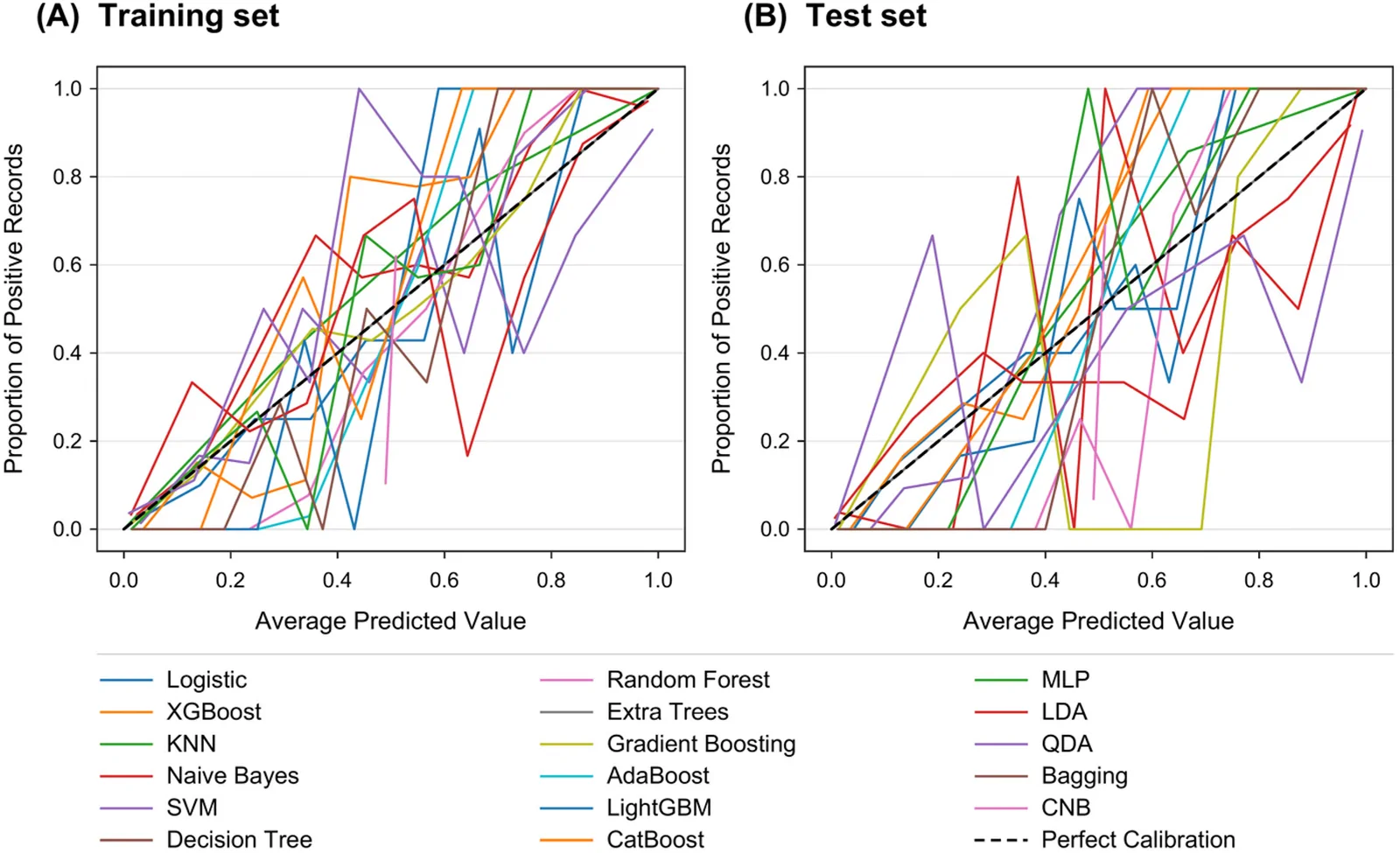
Calibration curves of machine learning–based prediction models for facial pressure injury in patients with chronic obstructive pulmonary disease receiving noninvasive positive pressure ventilation. **(A)** Training set and **(B)** Test set.

**Figure 4:**
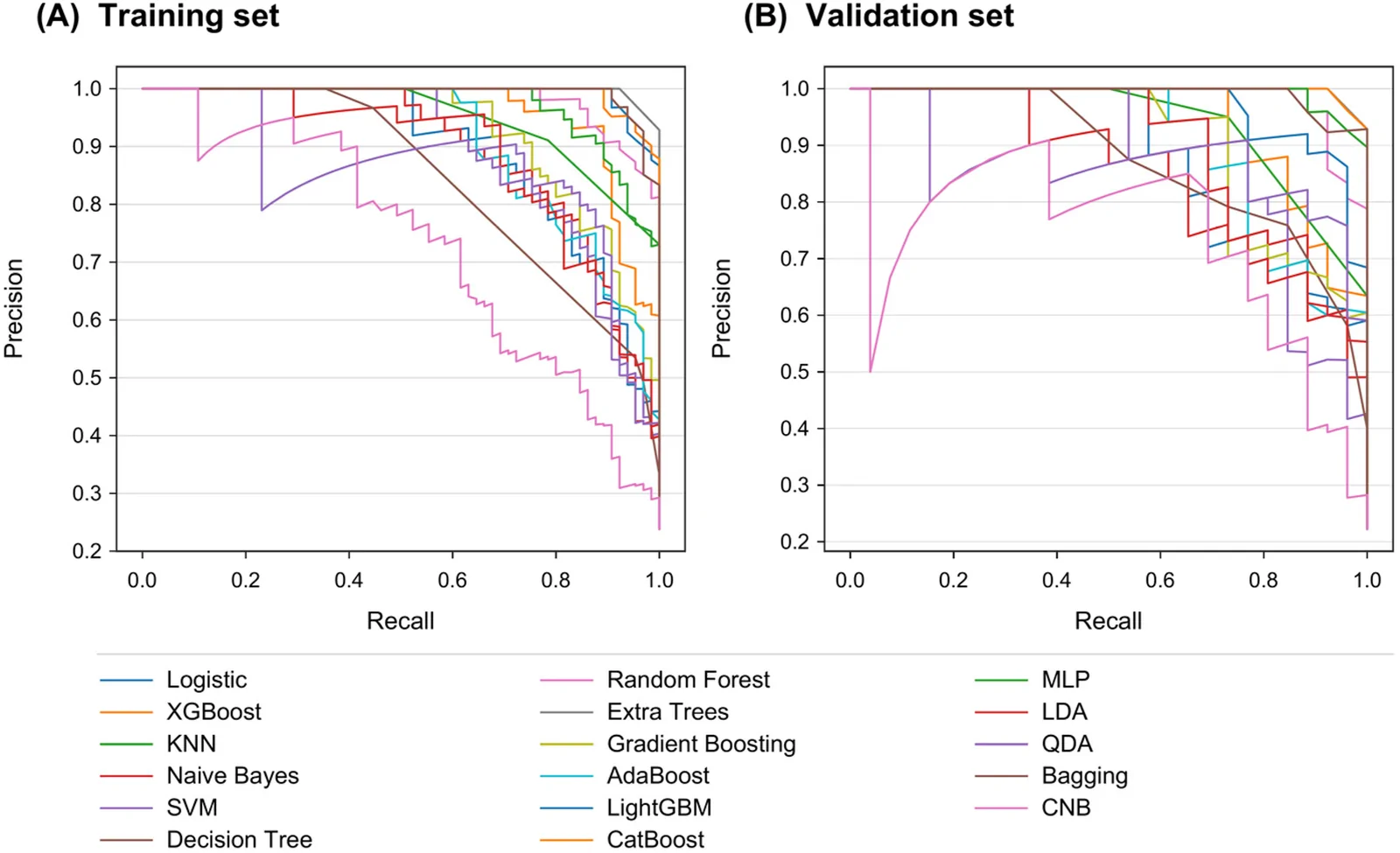
Precision–recall curves of machine learning–based prediction models for facial pressure injury in patients with chronic obstructive pulmonary disease receiving noninvasive positive pressure ventilation. **(A)** Training set and **(B)** Validation set.

**Figure 5:**
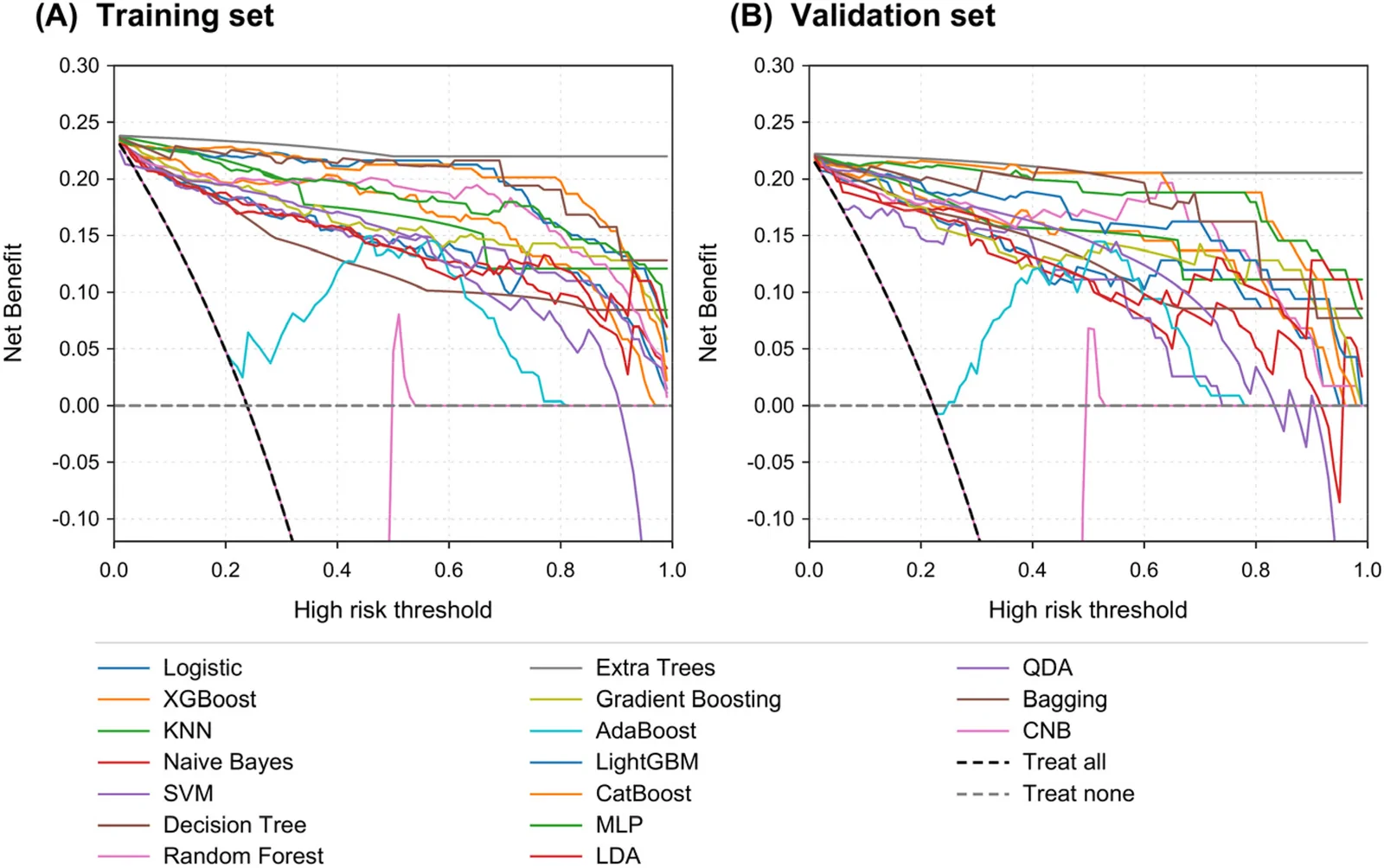
Decision curve analysis curves of machine learning–based prediction models for facial pressure injury in patients with chronic obstructive pulmonary disease receiving noninvasive positive pressure ventilation. (A) Training set and (B) Validation set.

### 3.5 Model evaluation

To compare the classification performance of different machine learning models for predicting facial pressure injury in patients with chronic obstructive pulmonary disease receiving noninvasive positive pressure ventilation, we conducted a comprehensive evaluation based on ROC and precision–recall curves as well as multiple metrics reported in Table 5. In the temporal validation cohort, AUC values ranged from 0.626 to 0.807. AdaBoost achieved the highest numerical AUC (0.807), followed by KNN (0.800), while logistic regression achieved an AUC of 0.785. Model selection considered both predictive performance and clinical applicability. Given its interpretability, simplicity, and ease of clinical implementation, logistic regression was selected as the final model and presented as a nomogram to facilitate bedside risk assessment in nursing practice (Figure 6). To illustrate the practical use of the nomogram, consider a hypothetical patient with an IPAP of 16 cm H₂O, a leak volume of 30 L/min, a BMI of 22 kg/m^2^, a total NPPV duration of 70 h, a single-session duration of 5 h, a serum albumin level of 38 g/L, and no history of NIV use. Reading vertically from each predictor value to the Points axis gives approximately 57, 17, 20, 27, 50, 0, and 0 points, respectively. The total score is approximately 171 points, corresponding to a predicted probability of facial pressure injury of approximately 30%. This example demonstrates how individual predictor values are converted into points, summed, and translated into an estimated probability.

**Table 5:** Comparison of multiple machine learning models for predicting facial pressure injury in patients with chronic obstructive pulmonary disease receiving noninvasive positive pressure ventilation.

Model name	AUC	F1	Specificity	Sensitivity	Youden index	*P* value
Training Set						
Logistic	0.886	0.732	0.947	0.677	0.624	Reference
XGBoost	0.873	0.757	0.966	0.677	0.643	0.008
KNN	0.866	0.761	0.961	0.692	0.654	0.003
NaiveBayes	0.864	0.740	0.962	0.662	0.623	0.044
SVM	0.903	0.745	0.932	0.723	0.656	0.032
DecisionTree	0.874	0.610	0.904	0.585	0.489	0.001
RandomForest	0.880	0.699	0.966	0.600	0.566	0.047
ExtraTrees	0.865	0.739	0.932	0.708	0.640	0.000
Gradient Boosting	0.904	0.729	0.937	0.692	0.630	0.014
AdaBoost	0.896	0.744	0.937	0.708	0.645	0.035
LightGBM	0.888	0.708	0.918	0.692	0.610	0.003
CatBoost	0.876	0.784	0.947	0.754	0.701	0.002
MLP	0.890	0.741	0.937	0.708	0.645	0.042
LDA	0.903	0.758	0.942	0.723	0.665	0.040
QDA	0.865	0.725	0.933	0.692	0.625	0.005
Bagging	0.864	0.742	0.938	0.708	0.645	0.008
CNB	0.814	0.644	0.865	0.677	0.542	0.001
Validation Set						
Logistic	0.785	0.642	0.945	0.580	0.525	Refence
XGBoost	0.730	0.642	0.945	0.580	0.525	0.082
KNN	0.800	0.737	0.967	0.660	0.627	0.050
NaiveBayes	0.745	0.680	0.923	0.680	0.603	0.181
SVM	0.743	0.589	0.901	0.580	0.481	0.087
DecisionTree	0.775	0.542	0.945	0.500	0.445	0.034
RandomForest	0.727	0.633	0.967	0.540	0.507	0.344
ExtraTrees	0.768	0.583	0.912	0.547	0.458	0.013
GradientBoosting	0.752	0.629	0.935	0.580	0.515	0.169
AdaBoost	0.807	0.614	0.901	0.613	0.514	0.064
LightGBM	0.785	0.567	0.901	0.540	0.441	0.035
CatBoost	0.693	0.653	0.923	0.620	0.543	0.058
MLP	0.649	0.606	0.878	0.620	0.498	0.063
LDA	0.722	0.640	0.912	0.620	0.532	0.197
QDA	0.626	0.529	0.934	0.473	0.407	0.003
Bagging	0.782	0.566	0.890	0.580	0.470	0.012
CNB	0.709	0.686	0.835	0.605	0.440	0.060

**Figure 6:**
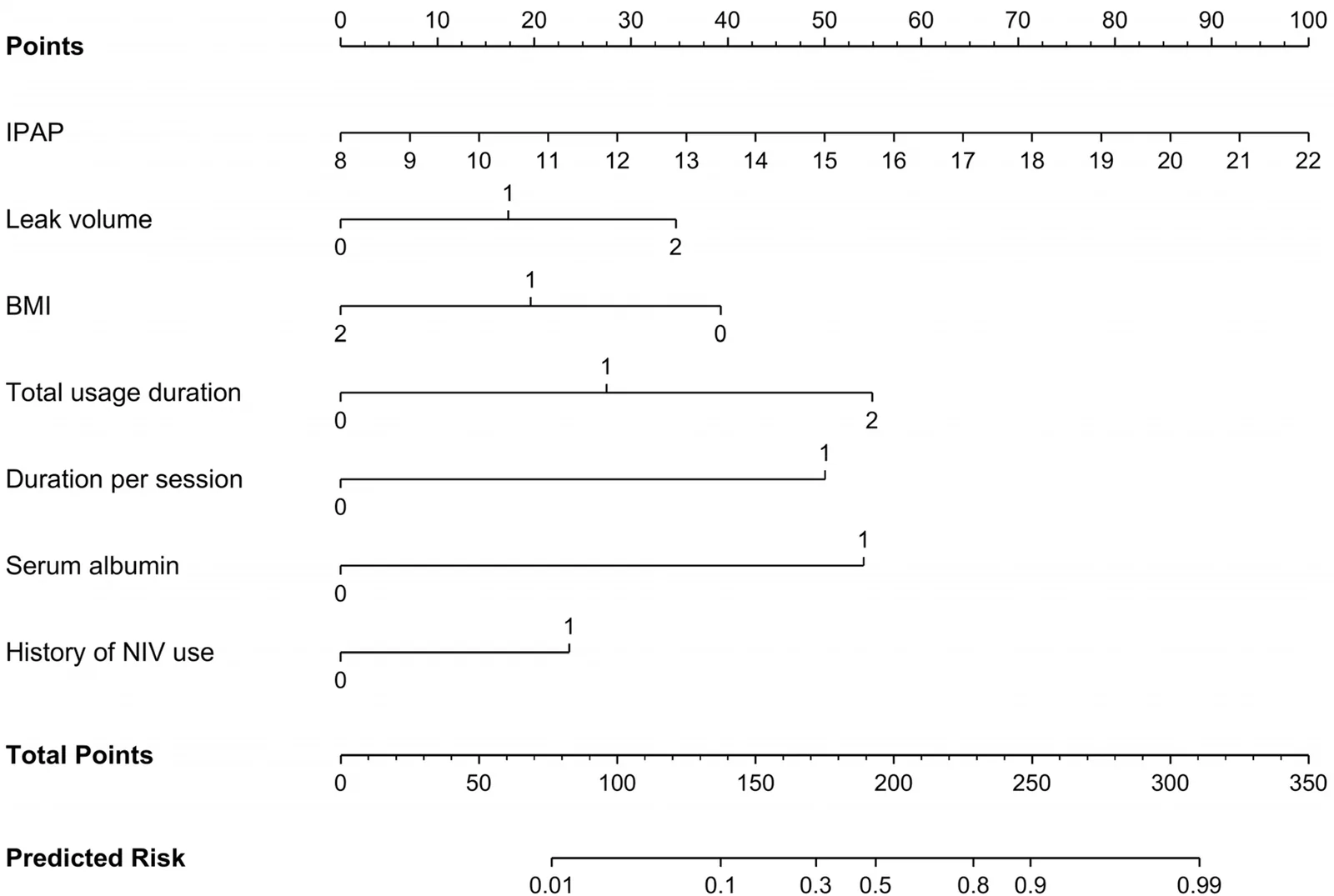
Nomogram of the logistic regression model for predicting facial pressure injury in patients with chronic obstructive pulmonary disease receiving noninvasive positive pressure ventilation.

### 3.6 SHAP interpretability analysis

To further interpret the prediction mechanism of the logistic regression model, SHAP was applied to quantify the contribution of each feature. Figure 7A presents the feature importance bar plot, and Figure 7B shows the SHAP beeswarm plot, illustrating the direction and magnitude of each variable's effect on the model output. The results indicated that serum albumin, total duration of NPPV use, and IPAP were the most influential features, followed by single-session duration and history of NIV use, with BMI and leak volume contributing to a lesser extent. The beeswarm plot further showed that hypoalbuminemia, longer total duration of NPPV use, higher IPAP levels, and single-session duration ≥4 h tended to increase the predicted risk of facial pressure injury. In contrast, higher BMI appeared to be associated with a lower predicted risk.

**Figure 7:**
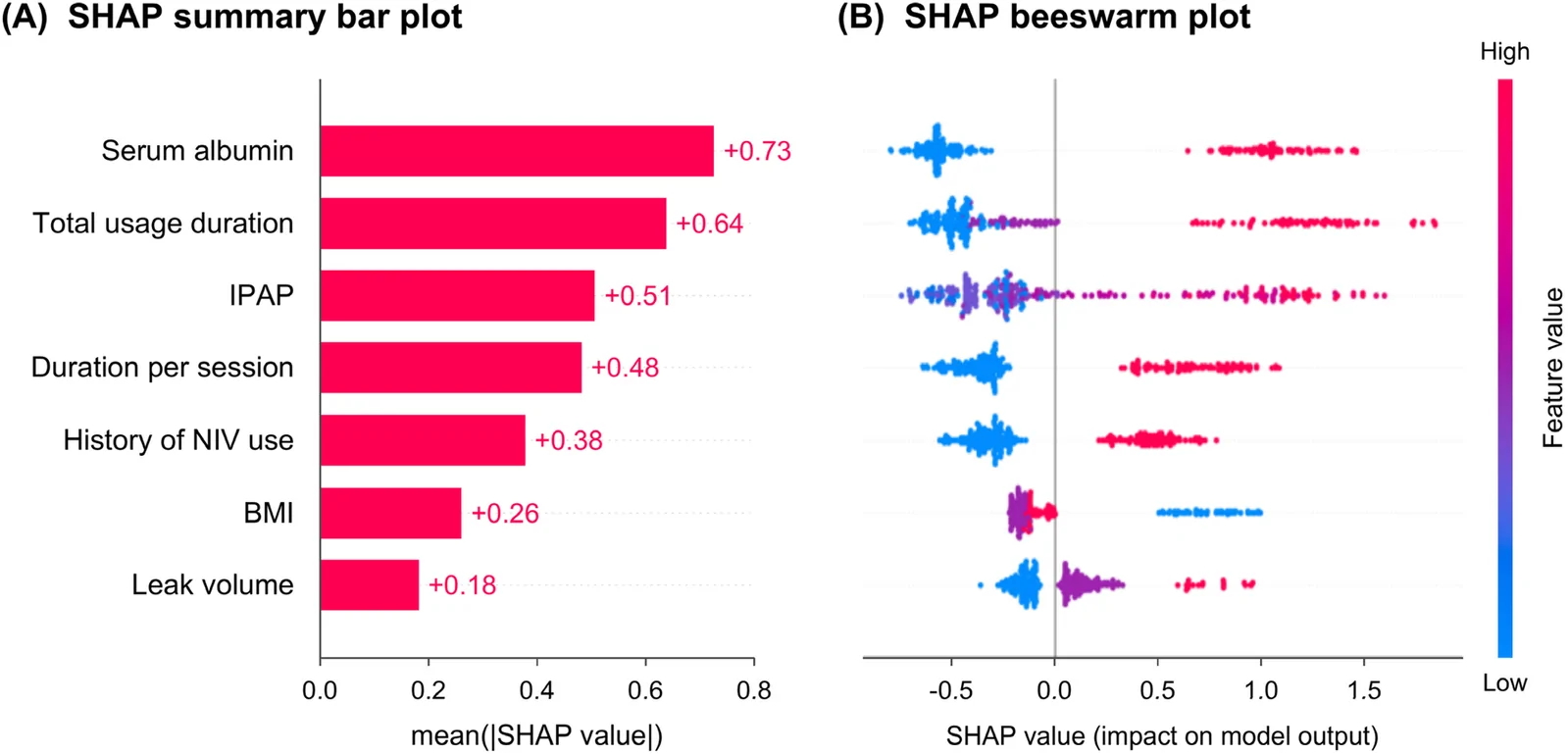
SHAP analysis of the logistic regression model for predicting facial pressure injury in patients with chronic obstructive pulmonary disease receiving noninvasive positive pressure ventilation. **(A)** SHAP summary bar plot and **(B)** SHAP beeswarm plot.

## 4 Discussion

### 4.1 High incidence of facial pressure injury and insufficient attention in clinical nursing practice

In this study, the incidence of NPPV-related facial pressure injury was 23.3%, which is consistent with the rate of device-related pressure injury associated with NPPV reported in a systematic review by Emami Zeydi et al. (approximately 20%–30%) (7) and markedly higher than the 7%–10% reported among general hospitalized patients (9). Sousa et al. (22) also confirmed in an ICU population that COPD is associated with an increased risk of device-related pressure injury. Nevertheless, clinical practice still largely relies on generic risk assessment tools such as the Braden and Norton scales (23). These instruments were primarily designed to assess overall pressure ulcer risk in bedridden patients and do not adequately incorporate NPPV-specific mechanical exposures, such as IPAP, leak volume, and single-session duration, leading to inherent limitations in early identification of device-related injury. In routine nursing care, early-stage injury may present only as indentation, erythema, or mild pain and can be misinterpreted as a transient pressure response, delaying timely intervention (24). Moreover, nursing staff often focus on ventilation efficacy indicators such as oxygenation improvement, while quantitative assessment and continuous monitoring of the relationship between ventilator parameters and skin injury remain insufficient. The latest AARC clinical practice guideline has explicitly emphasized the need for systematic assessment of the skin around NIV interfaces (25). In addition, common practices such as tightening the mask to reduce leaks and prolonging single-session duration to maintain ventilation may further increase the risk of local ischemia and shear-related tissue damage. Therefore, there is an urgent need to develop an NPPV-specific risk assessment tool that integrates key mechanical parameters with individual susceptibility factors, enabling early identification of high-risk patients and implementation of risk-stratified prevention while maintaining ventilation efficacy.

### 4.2 Phenotypic factors associated with facial pressure injury in patients with chronic obstructive pulmonary disease receiving noninvasive positive pressure ventilation

Our findings suggest that nutritional status, body habitus, and prior device exposure are key phenotypic risk factors for facial pressure injury during NPPV in patients with COPD. Notably, serum albumin <35 g/L emerged as the most influential risk factor in the SHAP analysis. Previous studies have likewise shown that hypoalbuminemia significantly increases the risk of pressure injury (26). In a machine learning study predicting pressure injury stage among ICU patients, Kahveci et al (27).also identified albumin level as a key predictor of severity and observed a trend toward lower albumin and higher inflammatory markers (e.g., C-reactive protein) with more advanced stages. Mechanistically, decreased albumin often reflects malnutrition and inflammatory stress, which can reduce plasma colloid osmotic pressure, promote tissue edema, weaken soft-tissue cushioning, and impair skin perfusion; it can also inhibit collagen synthesis and tissue repair, making microdamage under sustained mask pressure more likely to progress to overt pressure injury (28–30).

In addition, BMI <18.5 kg/m^2^ was associated with a markedly higher risk of injury. Multiple studies have confirmed that underweight status is an independent risk factor for pressure injury (31–33). Biomechanical evidence also indicates an inverse relationship between BMI and interface pressure, suggesting that lean patients are more likely to exceed the capillary closing pressure threshold, resulting in compromised local perfusion (34, 35). Interestingly, we also found that patients with a history of NPPV use were more prone to facial pressure injury. This may indicate more frequent or prolonged device exposure, or reflect recurrent disease exacerbations with greater nutritional depletion and reduced tissue tolerance (36), thereby increasing susceptibility. For example, Pittman et al. (37) identified a prior history of pressure injury as an important risk factor in critically ill patients. Moreover, studies by Liu et al. (10) and Lin et al. (38)reported that longer exposure time to noninvasive ventilation is associated with a higher risk of pressure injury, supporting a link between prior interface use and subsequent injury risk. A plausible mechanism is that repeated or prolonged mask wearing may cause subclinical soft-tissue damage and lower the tolerance threshold, while recurrent exacerbations and nutritional consumption further amplify the likelihood of injury. Therefore, serum albumin <35 g/L (39), BMI <18.5 kg/m^2^, and a history of NPPV use (40) should be considered key warning signals, prompting early nutritional support, interface pressure relief strategies, and dynamic skin assessment at the initiation of NPPV to reduce the incidence of facial pressure injury.

### 4.3 Biobehavioral factors associated with facial pressure injury in patients with chronic obstructive pulmonary disease receiving noninvasive positive pressure ventilation

During NPPV, ventilator settings and patterns of use directly determine the level of interface pressure and therefore constitute key biobehavioral factors for facial pressure injury. An important finding of this study is that higher IPAP was independently associated with facial pressure injury and was one of the most influential mechanical variables in the SHAP analysis. Lauderbaugh et al. (41) also reported a significant association between maximum inspiratory pressure and pressure injury in pediatric NPPV users, supporting IPAP as a critical risk factor across age groups. Pressure-mapping studies further suggest that the nasal bridge may be exposed to elevated interface pressure during NPPV, depending on ventilator settings and mask fit (42). Current understanding of pressure injury has expanded beyond a purely ischemic mechanism to include cellular deformation, ischemia–reperfusion injury, and impaired lymphatic drainage (8). The classic pressure–time relationship also suggests that the greater the pressure, the shorter the duration of tissue tolerance (43). However, in routine clinical nursing practice, attention to ventilator mechanical parameters remains insufficient. Under continuous ventilation, higher IPAP is more likely to act synergistically with prolonged use, accelerating local tissue damage. In addition, we found that leak volume >40 L/min significantly increased the risk of injury. Emami Zeydi et al. (7) have identified leak adjustment as a core strategy for preventing NPPV-related pressure injury. Quality-improvement studies also show that setting a target leak range can significantly reduce pressure injury incidence (44). Increased leak typically indicates poor mask fit or inadequate interface sealing. In practice, leaks are often corrected by tightening straps to improve fit. However, quantitative studies suggest that tightening may increase nasal-bridge interface pressure, potentially contributing to tissue injury, particularly in the presence of shear forces (8). Therefore, leak volume is not only an indicator of ventilation effectiveness but also an early signal of interface pressure imbalance.

In addition, this study showed that a total usage duration >100 h and a single-session duration ≥4 h were both strongly associated with injury occurrence (45). This is consistent with the findings of O’Dowd et al. (46), who reported an association between longer protective mask wear and an increased risk of pressure injury. Sustained pressure may impair local perfusion and contribute to ischemia and hypoxia. Reperfusion following pressure relief may be accompanied by oxidative stress and inflammatory responses, with repeated episodes potentially contributing to cumulative tissue damage (47), further supporting the synergistic effect of pressure and time (48). Collectively, higher IPAP, increased leak, and prolonged use constitute key modifiable behavioral risks for NPPV-related facial pressure injury. Clinically, ventilator pressure settings and fitting strategies should be optimized while ensuring ventilation efficacy, avoiding excessive strap tightening, limiting continuous wear time, and implementing intermittent pressure relief and interface rotation to reduce tissue load and the incidence of injury.

### 4.4 Clinical value and predictive performance of the model

In this study, 17 machine learning models were developed and compared based on seven core variables. DeLong's test showed that most complex algorithms did not achieve significantly better discrimination than logistic regression. Given the relatively limited sample size, these findings suggest that complex models may be more prone to overfitting in small-sample settings, whereas simpler and more interpretable traditional models are more suitable for clinical implementation. Therefore, logistic regression was prioritized as the recommended model for practical application. The model achieved an AUC of 0.785, an F1 score of 0.642, a sensitivity of 0.580, and a specificity of 0.945, indicating good overall discrimination with a low false-positive rate. The calibration curve demonstrated good agreement between predicted probabilities and observed incidence, suggesting acceptable calibration.

Regarding interpretability, SHAP analysis identified serum albumin, total usage duration, and IPAP as the top three contributors, consistent with the multivariable regression results. In the beeswarm plot, high-risk values were mainly distributed in the positive contribution region, indicating that the direction of risk aligned with the regression estimates. The nomogram based on the seven variables enables rapid bedside quantification of individual risk, facilitating real-time nursing risk stratification and decision-making. Compared with generic tools such as the Braden scale, this model incorporates NPPV-specific mechanical parameters and is therefore closer to the underlying etiological pathway, which may improve identification of high-risk patients and provide an actionable quantitative basis for risk-stratified prevention and resource allocation for facial pressure injury.

### 4.5 Implications and recommendations for clinical nursing practice

The seven independent risk factors identified in this study indicate that NPPV-related facial pressure injury is not an incidental event but rather the result of the combined effects of baseline susceptibility and treatment-related exposure. Because this model relies only on routinely available clinical data, it can be applied without additional tests or equipment, offering good scalability and potential health-economic advantages. Previous cost analyses have shown that preventing one pressure injury can save approximately USD 2,000–3,000 in medical costs (49). Therefore, we recommend incorporating NPPV-specific risk assessment into nursing quality-sensitive indicator systems to promote quantified management and risk-stratified prevention through standardized pathways.

To translate these risk factors into actionable measures, we propose the following recommendations based on our findings. First, for patients with serum albumin <35 g/L, initiate early nutritional assessment and consultation, provide high-protein support (e.g., ≥1.2 g/kg/day), and monitor relevant indicators dynamically (50). Second, incorporate IPAP, leak volume, and treatment duration into standardized process management. Set leak volume >40 L/min as an early warning threshold, and prioritize addressing leakage by optimizing mask type and fit rather than excessively tightening straps; routinely apply foam dressings to high-risk areas such as the nasal bridge and zygomatic regions and consider interface rotation to redistribute pressure (7, 51, 52). Third, in line with ISCCM guidance (53), implement modular NPPV training and use OSCE-based assessment to ensure consistency of clinical skills (54). Fourth, establish a multidisciplinary collaboration mechanism involving respiratory therapists, dietitians, wound care specialists, and primary nurses, with regular case reviews and quality feedback to form a closed-loop management process (55, 56). Fifth, strengthen patient and family education to help them recognize early warning signs and report them promptly, and discourage unassisted mask adjustment (57, 58). Sixth, at the implementation level, embed the prediction model into the nursing information system to complete baseline assessment within 24 h of admission, automatically push targeted interventions and document their execution, and incorporate overdue items into quality control follow-up, thereby reducing the incidence of facial pressure injury through a standardized pathway.

### 4.6 Strengths and limitations

This study is the first to use the SSM 2.0 as a theoretical framework to systematically integrate baseline patient characteristics, treatment exposure, and respiratory mechanical parameters and to develop a dedicated prediction model for NPPV-related facial pressure injury in patients with COPD. Compared with generic tools such as the Braden scale, our model addresses the limited sensitivity of universal instruments in the NPPV context and better captures heterogeneity in respiratory mechanics and treatment exposure. Methodologically, we performed temporal temporalvalidation to assess generalizability, developed and compared 17 algorithms, and applied SHAP for interpretable modeling. The results showed that, given the current sample size, complex algorithms did not provide meaningful incremental benefit. We therefore selected a parsimonious and transparent logistic regression model and presented it as a nomogram for bedside use. SHAP visualization further clarified the contribution and direction of key variables, improving interpretability and aligning with international guideline recommendations emphasizing device-specific risk assessment.

Several limitations should be noted. First, this was a retrospective study, and some relevant variables may not have been fully captured from medical and nursing records. Second, we did not include more direct mechanical indicators, such as mask type or interface pressure mapping, which may have reduced the precision of mechanical exposure assessment. Third, although the model was evaluated in a temporally separated validation cohort, both cohorts were derived from the same regional clinical setting. Therefore, this should be regarded as temporal validation rather than geographically independent external validation. Future studies should validate and update the model in larger, multicenter, geographically diverse, and prospective populations.

## 5 Conclusion

Guided by the SSM 2.0, this study integrated baseline susceptibility and NPPV-related treatment exposure to develop an individualized risk prediction tool for facial pressure injury in patients with COPD. We demonstrated that nutritional status, body habitus, prior ventilation exposure, and ventilator-related parameters including inspiratory pressure, leak, and duration of use are associated with injury risk. The model showed stable performance in temporalvalidation, supporting rapid clinical risk stratification and early warning in nursing practice. Ventilator parameters are not only indicators of ventilation efficacy but also early signals of interface pressure imbalance and skin risk. We recommend strengthening nurses’ competence in parameter interpretation and standardized adjustment, and incorporating parameter monitoring, fit optimization, pressure-relief protection, and follow-up education into standardized care pathways to achieve precise prevention and quality improvement of facial pressure injury in patients with COPD.The potential benefit of reducing facial pressure injury incidence requires confirmation in future implementation studies.

## Data Availability

The original contributions presented in the study are included in the article/Supplementary Material, further inquiries can be directed to the corresponding author.
